# Amplification efficiency and thermal stability of qPCR instrumentation: Current landscape and future perspectives

**DOI:** 10.3892/etm.2015.2712

**Published:** 2015-08-25

**Authors:** KARLY-RAI ROGERS-BROADWAY, EMMANOUIL KARTERIS

**Affiliations:** Department of Biosciences, Brunel University, Uxbridge UB8 3PH, UK

**Keywords:** quantitative polymerase chain reaction, thermal uniformity, amplification efficiency

## Abstract

Quantitative polymerase chain reaction (qPCR) is a method of amplifying and detecting small samples of genetic material in real time and is in routine use across many laboratories. Speed and thermal uniformity, two important factors in a qPCR test, are in direct conflict with one another in conventional peltier-driven thermal cyclers. To overcome this, companies are developing novel thermal systems for qPCR testing. More recently, qPCR technology has developed to enable its use in point-of-care testing (POCT), where the test is administered and results are obtained in a single visit to a health provider, particularly in developing countries. For a system to be suitable for POCT it must be rapid and reliable. In the present study, the speed and thermal uniformity of four qPCR thermal cyclers currently available were compared, two of which use the conventional peltier/block heating method and two of which use novel heating and cooling methods. The time required to complete 40 cycles varied between 12 and 58 min, and the C_t_ values were comparable, ranging between 13.6 and 16.8. Therefore, the novel technologies investigated in the present study for qPCR instrumentation performed equally well compared with conventional qPCR instruments, in terms of amplification efficiency and thermal uniformity.

## Introduction

Quantitative polymerase chain reaction (qPCR), the method by which a small sample of genetic material can be exponentially amplified and quantitatively measured in real time, is now a mainstay of research and medical laboratories. As the process has evolved, the applications for qPCR have increased rapidly, and include the detection of infectious diseases, paternity identification, forensic analysis and food processing. The PCR process necessitates the cycling of test samples through a temperature profile, typically 95, 55 and 72°C, multiple times. The time taken to change the temperature of the samples between these levels is a key determinant of the speed of the process and thus of the duration of a test (1). A typical 40-cycle PCR can take ~2 h to complete and improvements in that time have not been achieved as rapidly as the advances in other areas. Therefore, some of the potential benefits of the qPCR process remain limited by speed.

Thermal uniformity, the absence of which can cause discrepancies in the cycling conditions between different samples on the same plate, is directly linked to speed. In many PCR instruments, conductive blocks are used to connect the heating or cooling source(s) to the test samples. When heating and cooling the system, it is necessary to drive heat into and out of the block. Temperature gradients are eliminated by the natural flow of heat within the blocks; therefore, over time the same conditions should be delivered to all the test samples. However, block-based systems are vulnerable to greater heat losses on the edges and surfaces that tend to distort the thermal distribution. The conductivity of the blocks affects the rate of heat flow and thus the uniformity of heating of the samples. In addition, the larger the thermal mass of the block, the greater the amount of heat to be transferred and the longer this will take. The faster heat is driven into or out of the system, the less time is available for the temperature distribution of the conductive block to even out and for thermal uniformity to be maintained. Ultimately, such a system can only maintain thermal uniformity if the rate of change of temperature is slower than the time it takes for the temperature of the conductive block to even out. To achieve quick cycle times, large temperature gradients are applied to the block, which can lead to the target temperatures of samples being over- or undershot. Thus, in these types of systems, the requirement for uniformity of temperature directly conflicts with the desire for speed; they are able to deliver one feature or the other but not both (2).

The ABI Prism 7900HT (Applied Biosystems, Thermo Fisher Scientific, Waltham, MA, USA) is perhaps the industry standard peltier/block-based thermal cycler. The CFX96 (Bio-Rad Laboratories, Inc., Hercules, CA, USA) provided an upgrade to the conventional system by reducing the thermal mass of the block. Alternatives to the block-based system have also been developed. The Rotor-Gene Q (Qiagen, Hilden, Germany) combines a centrifugal set-up with an air-based thermal system. Ensuring that samples are continuously rotated through heated air removes the edge effect to provide superior thermal uniformity. xxpress® (BJS Biotechnologies, Perivale, London, UK) employs a different system in which an ‘active heating’ method is combined with a block of low thermal mass, precisely controlling the amount and location of additional heating to avoid temperature discrepancies (Table I). The present study investigated and compared the efficiency and thermal uniformity of four of the qPCR thermal cyclers currently available that use the conventional block/peltier system or novel methods.

## Materials and methods

### 

#### qPCR

The expression of 18S rRNA in human genomic DNA was assessed and compared by qPCR using an ABI Prism 7900HT, a Bio-Rad CFX96 System, a Qiagen Rotor-Gene Q and a BJS Biotechnologies xxpress®. Human genomic DNA was purchased from Bioline (Meridian Bioscience, London, UK) and input in concentrations of 100, 10, 1, 0.1 and 0.01 ng/µl to give final concentrations of 5, 0.5, 0.05, 0.005 and 0.0005 ng/µl, generating a standard curve. Eukaryotic 18S rRNA gene primers were used as follows: forward, 3′-AAA CGG CTA CCA CAT CCA AG-5′ and reverse, 3′-CCT CCA ATG GAT CCT CGT TA-5′. SYBR FAST qPCR master mix (Kapa Biosystems, Wilmington, MA, USA) was used across all platforms using the following thermal profile: 20 sec hot start at 95°C followed by 40 cycles of 95°C for 1 sec and 60°C for 10 sec. Heating and cooling rates and all other parameters were at the manufacturers' pre-set levels.

Thermal variability was assessed in qPCR by measuring the amplification of 18S rRNA in a selection of wells covering all areas of the sample plate on ABI Prism 7900HT, Bio-Rad CFX96 System, Qiagen Rotor-Gene Q and BJS Biotechnologies xxpress instruments. Human genomic DNA at 100 ng/µl (final concentration, 5 ng/µl) was used with the protocol detailed above.

A standard curve was generated by amplifying 18S rRNA in human genomic DNA at concentrations of 5, 0.5, 0.05, 0.005 and 0.0005 ng/µl and plotting C_t_ against log concentration. Efficiency was calculated by the following equation: Efficiency = 10^(−1/slope)-1^. Efficiency of reaction values between 90 and 110% are considered acceptable for qPCR reactions.

#### Statistical analysis

Statistical tests commonly used to determine the reliability and accuracy of a quantitative PCR assay include performing a standard curve experiment with each dilution series run in triplicate. The C_t_ value was plotted against the log of the nucleic acid input level to generate a linear graph. The slope or gradient of this graph was used to determine the PCR reaction efficiency and a linear regression analysis with a correlation coefficient or R^2^ value was included to determine the accuracy and repeatability of the standard curve. The ideal result is a PCR reaction efficiency of 100% and an R^2^ value of 1. An efficiency of <90 or ≥110% is unacceptable and indicates that further optimisation is required. If the R^2^ value is ≤0.985, this raises questions about assay reliability with respect to pipetting accuracy and the range of the assay (3).

## Results

### 

#### Amplification efficiency and thermal variability

**Figure 1.**
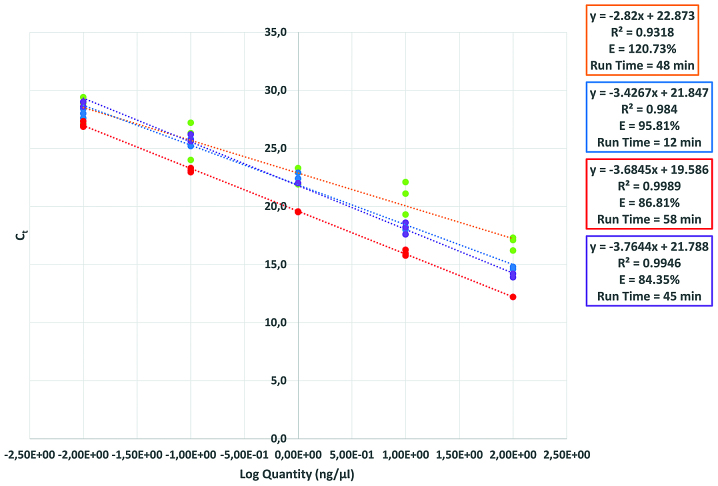
Amplification efficiency of four qPCR instruments; CFX96 (green), xxpress (blue), ABI Prism 7900 HT (red) and Rotor-Gene Q (purple).

**Figure 2.**
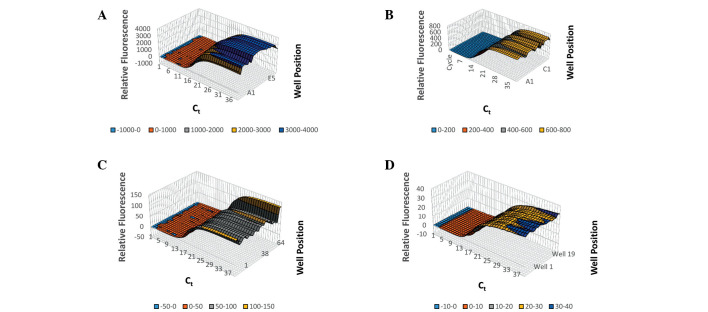
Thermal variability upon amplification of 18s rRNA using 5 ng/µl human genomic DNA. (A) CFX96, (B) xxpress®, (C) ABI Prism 7900HT and (D) Rotor-Gene Q.

**Figure 3.**
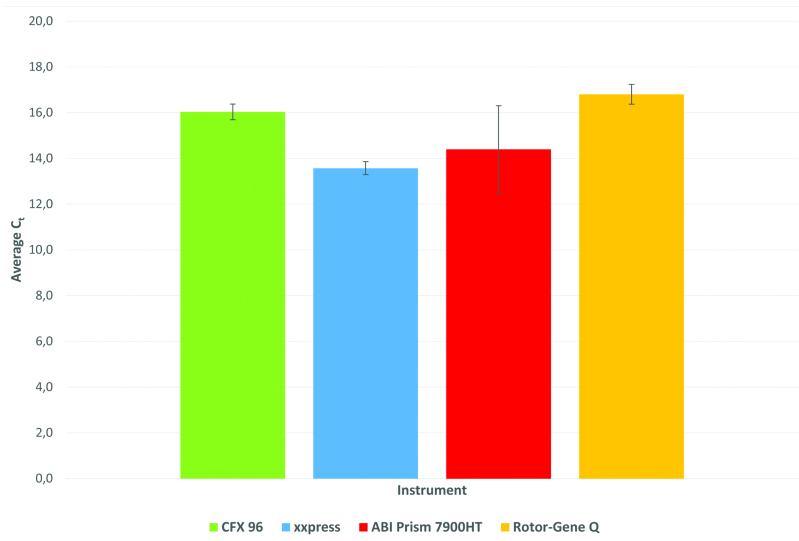
Average C_t_ of all instruments.

The fastest instrument was the xxpress®, which completed 40 cycles in 12 min (Fig. 1). In terms of amplification efficiency there was a variation of ≤36 min among the different cyclers (Fig. 1). The time required to complete a PCR run may be of particular interest to clinicians, as it may be used as a point of care testing platform. This is of increasing significance, particularly for diseases that currently require hours or days to diagnose. Thermal variability was assessed by measuring the amplification of 18S rRNA in 5 ng/µl human genomic DNA in a selection of wells covering all areas of the sample plate on ABI Prism 7900HT, Bio-Rad CFX96 System, Qiagen Rotor-Gene Q and BJS Biotechnologies xxpress instruments (Fig. 2). The average C_t_, C_t_ spread and C_t_ standard deviation were for CFX: 16.0, 1.315 and 0.34; for xxpress: 13.6, 1.2 and 0.29; for Prism 7900HT: 14.4, 4.526, and 1.91; and for Rotor-Gene: 16.8, 1.319, and 0.43 (Fig. 3).

## Discussion

qPCR instrumentation is rapidly evolving not only to meet the needs of basic science but also in an attempt to address some of the needs of the current healthcare system, in terms of diagnosis as well as prognosis. For example, qPCR technology has been widely used in the field of molecular diagnostics for a number of infectious diseases (4). Food and Drug Administration-approved qPCR-based screening tests include group A *Streptococcus* and methicillin-resistant *Staphylococcus aureus* (MRSA), HIV-1, human metapneumovirus and H1N1 influenza virus (5,6). More recently, Qiagen received FDA approval of a therascreen® KRAS RGQ PCR kit, paired with a colorectal cancer drug. KRAS mutations occur in ~40% of colorectal cancer patients (7,8). Therefore, screening patients by PCR will detect the most frequent mutations in the KRAS gene and should aid with the selection of therapeutic interventions.

Over the past decade, there has been a shift from testing in reference hospitals/centres to clinical/diagnostic laboratories worldwide (4). Point of care testing (POCT) allows a test to be carried out and results obtained in a single visit to a primary or secondary care health provider (9). In developing countries, POCT is perhaps even more effective. The requirement for expensive, central laboratories, highly trained technicians and a reliable method of specimen and data transport can all be removed with the implementation of a well-designed, multifunctional POCT system. Bringing the test into the clinic allows treatment to commence without delay and, in areas of high displacement, reduces the likelihood of losing patient contact before the condition has been effectively treated. This is particularly important for communicable diseases such as HIV/AIDS, measles and typhoid fever (10). An effective POC test in a low resource setting is inexpensive to use and maintain. The test must be easy to operate, requiring little to no training or specialist knowledge to both generate and interpret results. In a recent study of sub-Saharan Africa, only 34% of hospitals had reliable electricity access (11). Since energy access for healthcare facilities in this region varies markedly, and as electrical sources may be unreliable, low electrical consumption or even the ability to run on battery or solar power is desirable.

The results of the present study demonstrate that the performance of new technologies in qPCR instrumentation such as Rotor-Gene Q and xxpress, is equally as good as that of conventional qPCR instruments, in terms of amplification efficiency and thermal uniformity. Notably, an advantage of the new technologies is the fast delivery, as rapid testing and diagnosis may be lifesaving. For example, rapid diagnostic tests can help in the diagnosis and management of patients who present with signs and symptoms compatible with influenza. These technologies can reduce the time from 3–10 days for conventional viral cell cultures, and requires minutes rather than hours to perform, which may be of clinical benefit (12). Infections with MRSA are known to be associated with considerable morbidity and mortality (13). Current sample preparation/testing times based on blood samples can take up to 5 h. However, in an emergency situation this process might be too long if the patient admitted is positive for MRSA and therefore has the potential to infect others. Equally, an early diagnosis of tuberculosis will assist not only in the initiation of appropriate treatment but also limit the spread of this highly contagious disease (14). A test that could be administered either at admission to the clinic, or even in an ambulance on the way to the hospital, and takes only 10 min could be of real benefit. Moreover, given the unreliability of electricity in the developing world, diagnostic instrumentation that is rapid is vital.

**Figure 4.**
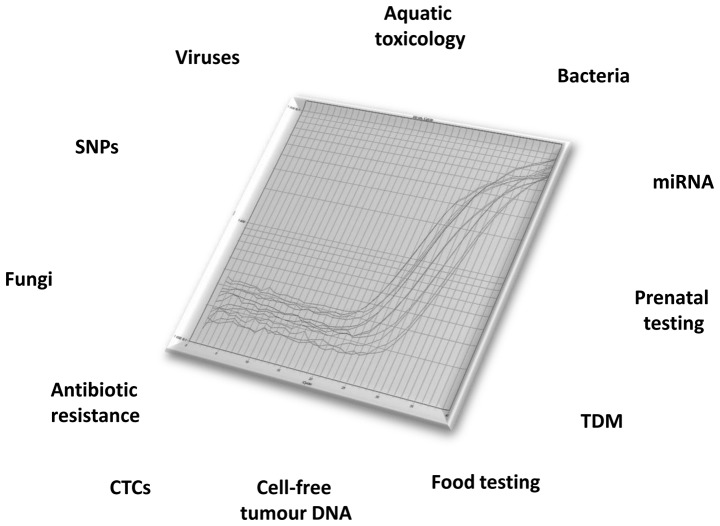
Current and future applications of qPCR testing. CTCs, circulating tumour cells; SNPs, single nucleotide polymorphisms; TDM, therapeutic drug monitoring; miRNA, microRNA.

To date, qPCR-based diagnosis is often associated with high cost, time-consuming procedures, scientists and clinicians trained in qPCR analysis, lack of specificity and sensitivity or even standardisation for certain tests. In the future (Fig. 4), a standardised, rapid, scalable, affordable and easy-to-use qPCR platform for use in POCT should provide an invaluable platform in the field of diagnostic/prognostic testing that will complement the current conventional methods, including microscopy, cell culture and immunological-based methods.

## Figures and Tables

**Table I.** Ramp rate and thermal uniformity of qPCR instruments.

qPCR platform	Thermal system	Advertised fastest ramp rate (°C/sec)	Advertised thermal uniformity (°C)
ABI Prism 7900HT	Block/peltier	1.5	±0.5 (measured 30 sec after timing starts)
Bio-Rad CFX96	Block/peltier	3.3 (average)	±0.4 (well-to-well within 10 sec of reaching 90°C)
Qiagen Rotor-Gene Q	Air	15 (peak)	±0.02
BJS biotechnologies xxpress®	Resistive heating	10	±0.3

qPCR, quantitative polymerase chain reaction.
